# Circulating miR-223 in Oral Cancer: Its Potential as a Novel Diagnostic Biomarker and Therapeutic Target

**DOI:** 10.1371/journal.pone.0159693

**Published:** 2016-07-21

**Authors:** Hirohiko Tachibana, Ri Sho, Yuji Takeda, Xuhong Zhang, Yukie Yoshida, Hiroto Narimatsu, Katsumi Otani, Shigeo Ishikawa, Akira Fukao, Hironobu Asao, Mitsuyoshi Iino

**Affiliations:** 1 Department of Dentistry and Oral and Maxillofacial Surgery, Yamagata University Faculty of Medicine, Yamagata, Japan; 2 Department of Public Health, Yamagata University Faculty of Medicine, Yamagata, Japan; 3 Department of Immunology, Yamagata University Faculty of Medicine, Yamagata, Japan; 4 Department of Biochemistry and Molecular Biology, Yamagata University Faculty of Medicine, Yamagata, Japan; SAINT LOUIS UNIVERSITY, UNITED STATES

## Abstract

Circulating microRNAs (miRNAs) have been detected in various types of cancer and have been proposed as novel biomarkers for diagnosis and treatment. Until recently, however, no studies had comprehensively examined circulating miRNAs in oral cancer. The current study used an ultra-sensitive genome-wide miRNA array to investigate changes in circulating miRNAs in plasma from five patients with oral cancer and ten healthy individuals. Results indicated that there were only a few circulating miRNAs, including miR-223, miR-26a, miR-126, and miR-21, that were up-regulated in patients with oral cancer. A subsequent validation test indicated that circulating miR-223 levels were significantly higher (~2-fold, P< 0.05) in patients with oral cancer (n = 31) than in those without cancer (n = 31). Moreover, miR-223 was found to be up-regulated in tumor-adjacent normal tissue compared to tumor tissue from patients with oral cancer. A gain-of-function assay was performed to explore the potential roles of circulating miR-223 in the development of oral cancer. Results revealed that miR-223 functions as a tumor suppressor by inhibiting cell proliferation and inducing apoptosis. In conclusion, this study suggested that circulating miR-223 may serve as a potential biomarker for diagnosis and that it may represent a novel therapeutic target for treatment of oral cancer.

## Introduction

Oral cancer, although uncommon, is increasingly more common in Japan due to aging of the population [1, 2]. The overall five-year survival rate (~55%) has not significantly changed yet because more than half of the cases of oral cancer are diagnosed as late-stage malignancy [3]. Although treatment options include surgery, radiotherapy, and chemotherapy, surgical removal of later stage cancer may require reconstruction of portions of the oral cavity or facial features and radiotherapy often causes serious adverse reactions [4]. Therefore, the pressing task is to gain a deeper understanding of molecular events involved in oral cancer and to search for new diagnostic biomarkers and therapeutic targets [5, 6].

MicroRNAs (miRNAs) are single-stranded small non-coding RNAs (ncRNAs) consisting of 19–24 nucleotides that negatively regulate the expression of their target mRNAs. Since miRNAs were first discovered in the nematode *C*. *elegans* in 1993 [7], more than 2,500 human miRNAs have been reported to date [8]. Numerous studies have indicated that miRNAs are involved in important biological processes such as cell proliferation, differentiation, and apoptosis, and that they are related to various diseases including cancer [9]. Evidence that miRNA expression differs between normal and cancer tissue and that this expression varies across different cancer types has opened up new avenues in cancer research [10]. In particular, a number of extracellular miRNAs were found to circulate in blood in a considerably stable form and their circulating profiles were found to be strongly associated with the progression of and prognosis for several types of cancer [11, 12], shedding light on miRNA-based cancer diagnostics and therapeutics. Elevated plasma levels of several miRNAs, including miR-184 [13], miR-24 [14], miR-31 [15], miR-10b [16], and miR-181 [17], have been reported in patients with oral cancer, and their potential clinical significance has been noted. Nevertheless, little is known about the circulating miRNA profiles in patients with oral cancer due in large part to the dearth of miRNAs in blood and the low efficacy of screening tests [18]. In addition, although extracellular circulating miRNAs have emerged as promising noninvasive biomarkers for oral cancer diagnosis, their origin and biological relevance still remain largely unclear [19].

The present study used a sensitivity high-throughput miRNA array to investigate and compare the plasma miRNA profiles of patients with oral cancer and healthy individuals. To the extent known, this is one of the first studies to measure genome-wide circulating miRNAs in oral cancer. A subsequent validation test revealed that miR-223 was up-regulated in plasma as well as in tumor-adjacent normal tissues from patients with oral cancer. Furthermore, an *in vitro* functional assay showed that miR-223 acts as tumor suppressor, suggesting that circulating miR-223 not only has the potential to serve as a biomarker but also plays a rather beneficial role in the pathology of oral cancer.

## Materials and Methods

### Clinical samples

Thirty-one patients with gingival squamous cell carcinoma (GSCC) (S1 Table) and 31 age- and sex-matched healthy controls were recruited from Yamagata University Hospital, Yamagata Prefectural Central Hospital, and Shonai Amarume Hospital (Yamagata, Japan) between February 2012 and April 2014. Blood plasma samples were obtained from patients and controls with their written informed consent under protocols approved by the Ethics Committee of the Yamagata University Faculty of Medicine (ethical approval 22–114). Samples were stored at -80°C until use. Twenty fresh sets of primary GSCC tumor tissues and adjacent normal tissues obtained during surgery were immediately immersed in RNAlater (Qiagen, Valencia, CA) and stored at -20°C until RNA was extracted. Cancer was diagnosed and classified according to the 2002 Union for International Cancer Control TNM staging criteria [20].

### Cell lines

The human oral squamous cell carcinoma (OSCC) cell lines SAS (#RCB1974), HSC-3 (#RCB1975), HSC-4 (#RCB1902), and Ca9-22 (#RCB1976) were purchased from RIKEN BRC Cell Bank (Tsukuba, Japan; 2013), where the cell lines were authenticated by STR profiling before distribution. Cells were cultured in Minimum Essential Medium (MEM, Invitrogen/GIBCO, Carlsbad, CA) supplemented with 10% fetal bovine serum (Nichirei Bio., Tokyo, Japan) in a humidified atmosphere containing 5% CO_2_ at 37°C according to supplier’s instructions. Cells were used within 6 months of resuscitation.

### RNA extraction

Total RNA used to determine the circulating miRNA profile was extracted from 1000 μl of pooled plasma from 5 patients with GSCC (200 μl/case) and 1000 μl of pooled plasma from 10 healthy controls (100 μl/individual). RNA was extracted using the 3D-Gene RNA extraction Kit (Toray, Kamakura, Japan) in accordance with the manufacturer’s instructions.

RNA used for quantitative real-time RT-PCR was isolated from 100 μl of a plasma sample or 10 μg of a tissue sample or 2 × 10^6^ cultured cells. RNA was isolated with the QIAGEN miRNeasy Kit in accordance with the manufacturer’s protocols.

### Determination of the circulating miRNA profile

Extracted total RNA was labeled with Hy5 using the miRCURY LNA Array miR labeling kit (Exiqon, Vedbaek, Denmark). Labeled RNAs were hybridized onto 3D-Gene Human miRNA Oligo chips (Toray, Kamakura, Japan). Annotation and oligonucleotide sequences of the probes conformed to the miRBase database (http://microrna.sanger.ac.uk/sequences/). After stringent washing, fluorescent signals were scanned with the 3D-Gene Scanner (Toray) and analyzed using 3D-Gene Extraction software (Toray). The microarray data is available on NCBI’s GEO database (Accession number: GSE81828).

The raw data for each spot were normalized to the mean intensity of background signals determined by all blank signal intensities at a 95% confidence interval. Measurements of spots with signal intensities greater than 2 standard deviations (SD) of the background signal intensity were considered to be valid. Relative levels of expression of a given miRNA were calculated by comparing the signal intensities of the valid spots throughout the microarray experiments. Normalized data were globally normalized on each array, such that the median of the signal intensity was adjusted to 25.

### Quantitative real-time RT-PCR

First-strand cDNA was synthesized from 5 μl of total RNA (plasma) or 10 ng of total RNA (tissue/cells) using the High-capacity cDNA Reverse Transcription Kit (Applied Biosystems Carlsbad, CA). Quantitative real-time PCR was carried out in the 7500 Fast Real-Time PCR System (Applied Biosystems). The initial PCR step consisted of a 10 min hold at 95°C, followed by 40 cycles of denaturation at 95°C for 15 sec and annealing/extension at 60°C for 1 min. Levels of expression of miRNAs, e.g. miR-223 (002295; Ambion, Applied Biosystems), were normalized to *let7a* (MC12169; Ambion) or RNU6B (001093; Ambion). The ΔΔCt method was used to calculate the relative quantity of the target miRNA [21]. All reactions were performed in triplicate and included negative control reactions that lacked cDNA.

### Pre-miRNA transfection

Unless otherwise stated, a total of 10 nM of Pre-miR hsa-miR-223 precursor (Pre-miR-223, 001093; Ambion) or Pre-miR negative control miRNA (Pre-miR-NC, AM17111; Ambion) was transfected with Lipofectamine RNAiMax reagent (Invitrogen, Carlsbad, CA) into 1 × 10^5^/ml of Ca9-22 cells in accordance with the manufacturer’s instructions. Cells were harvested and subjected to phenotypic and functional analysis 48 h after transfection. The amount of miR-223 in Ca9-22 cells was monitored with TaqMan real-time RT-PCR.

### Cell proliferation and viability assays

In order to measure cell proliferation and viability, Ca9-22 cells were seeded into 24-well plates (1.0 × 10^5^ cells/well) and transfected with either Pre-miR-223 or Pre-miR-NC at the specified concentrations. Cell samples were harvested at 24, 48, 72, 96, 120, and 144 h after transfection. The number of cells was determined by manual hemocytometer counting, and viability was assessed using the trypan blue exclusion method. Alternatively, cells seeded in 96-well plates (1.0 × 10^4^ cells/well) and transfected with the corresponding Pre-miRs. WST-1 colorimetric assay (Roche, Indianapolis, IN) were used for simultaneous measurement of cell proliferation and viability at various time points or with various quantities of Pre-miRNAs. Briefly, 10 μl of ready-to-use WST-1 solution was added directly to the cell culture in a 96-well plate. After incubation for 2 h, absorbance at 450 nm was measured using a microplate autoreader (BioRad, Hercules, CA) with a reference wavelength of 690 nm.

### Cell migration assay

After incubation at 37°C for 48 h, Ca9-22 cells transfected with either Pre-miR-223 or Pre-miR-NC were used in a migration assay. In brief, 1.0 × 10^5^ cells were plated in the upper chamber with a non-coated membrane (24-well insert; pore size 8 mm; BD Biosciences, Bedford, MA). MEM without FBS was placed in the upper chamber while MEM supplemented with 10% FBS was placed in the lower chamber. After incubation at 37°C for 18 h, non-migrating cells in the upper membrane surface were removed by careful wiping with a cotton swab. Cells that had passed through the membrane were stained using a Diff-Quik kit (Dade Behring, DE) and counted manually under a microscope. Triplicate wells were measured for cell migration in each treatment group.

### Measurement of apoptosis

After incubation at 37°C for 48 h, Ca9-22 cells transfected with either Pre-miR-223 or Pre-miR-NC were collected into microtubes with phosphate buffered saline (PBS, pH 7.4) containing 0.2% trypsin 250 (Difco, Detroit, MI) and 5-ethylenediaminetetraacetic acid (EDTA). After cells were washing with PBS, apoptotic cells were stained using the MEBCYTO Apoptosis Kit (MBL, Nagoya, Japan) in accordance with the manufacturer’s instructions, and measured using FACSCanto II (BD Biosciences, San Jose, CA).

### Western blotting

Ca9-22 cells were harvested for immunoblotting analysis 48 h after transfection of Pre-miR-223 or Pre-miR-NC. Cells were washed twice with PBS and then lysed in PBS containing 1% Triton X-100 and 1 × protease inhibitor. Equivalent amounts of cell lysates were separated by SDS polyacrylamide gel electrophoresis (SDS-PAGE) and then transferred to PVDF membranes. Immunoblotting was performed with STMN1 antibody (1:1000; #3352, Cell Signaling Technology, Tokyo, Japan) or IGF1R antibody (1:1000; #9750, Cell Signaling), and GAPDH (#3683, Cell Signaling) was used as an internal control. Membranes were washed and incubated with goat anti-rabbit IgG (H+L)-HRP conjugate (Bio-Rad, Hercules, CA). Specific complexes were visualized with ECL Plus™ (GE Healthcare Japan Co., Tokyo).

### Statistical analysis

Levels of miRNA expression in plasma or tissue were compared using a Student’s t test, analysis of variance (ANOVA), a non-parametric Mann-Whitney U-test, or a *χ*^*2*^-test. Receiver operating characteristics (ROC) curves were constructed to evaluate the diagnostic value of plasma miR-223 as a marker of oral cancer. A *P* value of less than 0.05 was considered to be statistically significant. Data are presented as the mean ± SD unless stated otherwise. All statistical analyses were performed using SPSS Statistics, Version 19.0 (IBM Corp., Armonk, NY).

## Results

### Circulating miRNA profile in patients with GSCC

**Fig 1:**
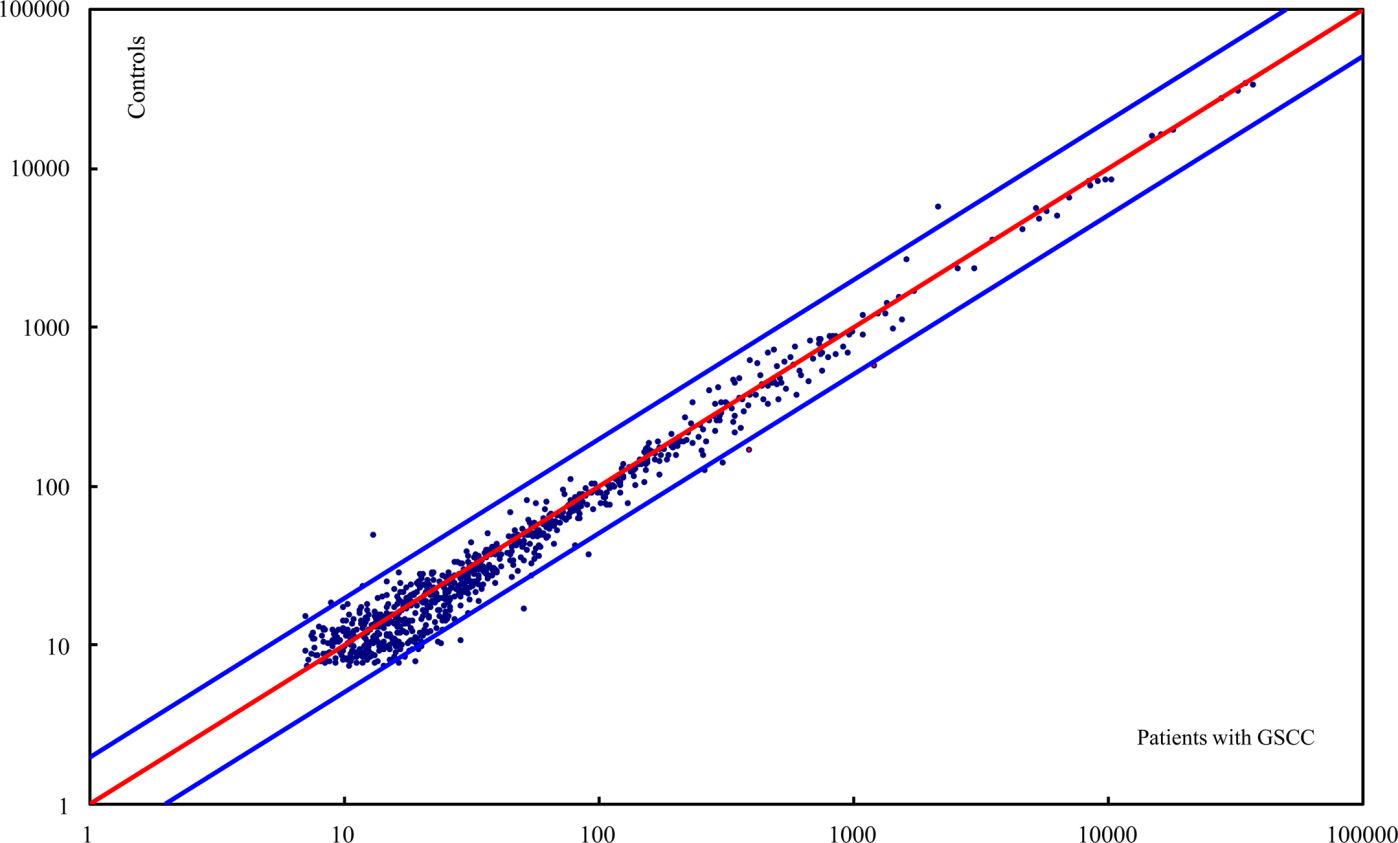
Plasma miRNA profile in patients with GSCC. The scatter plot compares the relative levels of expression of genome-wide miRNAs in plasma from patients with GSCC and healthy controls. Each point represents an individual miRNA. The red line represents a fold change of 1, and the blue lines indicate a 2-fold change in the level of miRNA expression in patients with GSCC (Top line: 2-fold down; Bottom line: 2-fold up).

Circulating levels of a total of 1,211 genome-wide miRNAs were measured with a microarray assay using pooled plasma samples from 5 patients with GSCC and pooled normal plasma samples from 10 age- and sex-matched healthy controls. The circulating miRNA profile for patients with oral cancer was compared with that for controls. Among the 854 detectable miRNAs, most varied little between patients with oral cancer and controls whereas 16 miRNAs were considerably up-regulated (> a 2-fold change) and 4 miRNAs were considerably down-regulated (> a 2-fold change) in patients with GSCC (Fig 1). As shown in Table 1, 4 of the 16 miRNAs that were highly expressed in patients with GSCC (miR-26a, miR-126, miR-223, and miR-21) were present in relatively large amounts in plasma from both cancer patients and controls. Given that miR-223 resulted in the most abundant circulating miRNA and that dysregulation of miR-223 has been noted in cancers originating from the lung, and colon [22, 23], miR-223 was chosen for study in further detail.

**Table 1:** Up-regulated miRNAs obtained by comparing patients with GSCC and controls.

**miRNAs**	**Signal intensity**		**Fold change**
	**Patients with GSCC**	**Controls**	**Patients with GSCC/Controls**
**miR-26b**	**51.3**	**16.7**	**3.07**
**miR-143**	**28.7**	**10.6**	**2.71**
**miR-199a-3p**	**91.7**	**37.1**	**2.47**
**miR-132-5p**	**19.1**	**7.9**	**2.42**
**miR-140-5p**	**24.2**	**10.3**	**2.36**
**miR-26a**	**389.9**	**171.4**	**2.27**
**miR-372**	**23.5**	**10.4**	**2.27**
**miR-126**	**308.4**	**138.9**	**2.22**
**miR-3927**	**16.4**	**7.7**	**2.14**
**miR-223**	**1208.8**	**572.6**	**2.11**
**miR-21**	**260.1**	**125**	**2.08**
**miR-644**	**19.5**	**9.4**	**2.07**
**miR-200c**	**20.1**	**9.7**	**2.07**
**miR-129-3p**	**17.4**	**8.5**	**2.05**
**miR-27a**	**54.8**	**27.1**	**2.03**
**miR-1263**	**17.4**	**8.7**	**2.01**

### A higher level of circulating miR-223 in patients with GSCC

**Fig 2:**
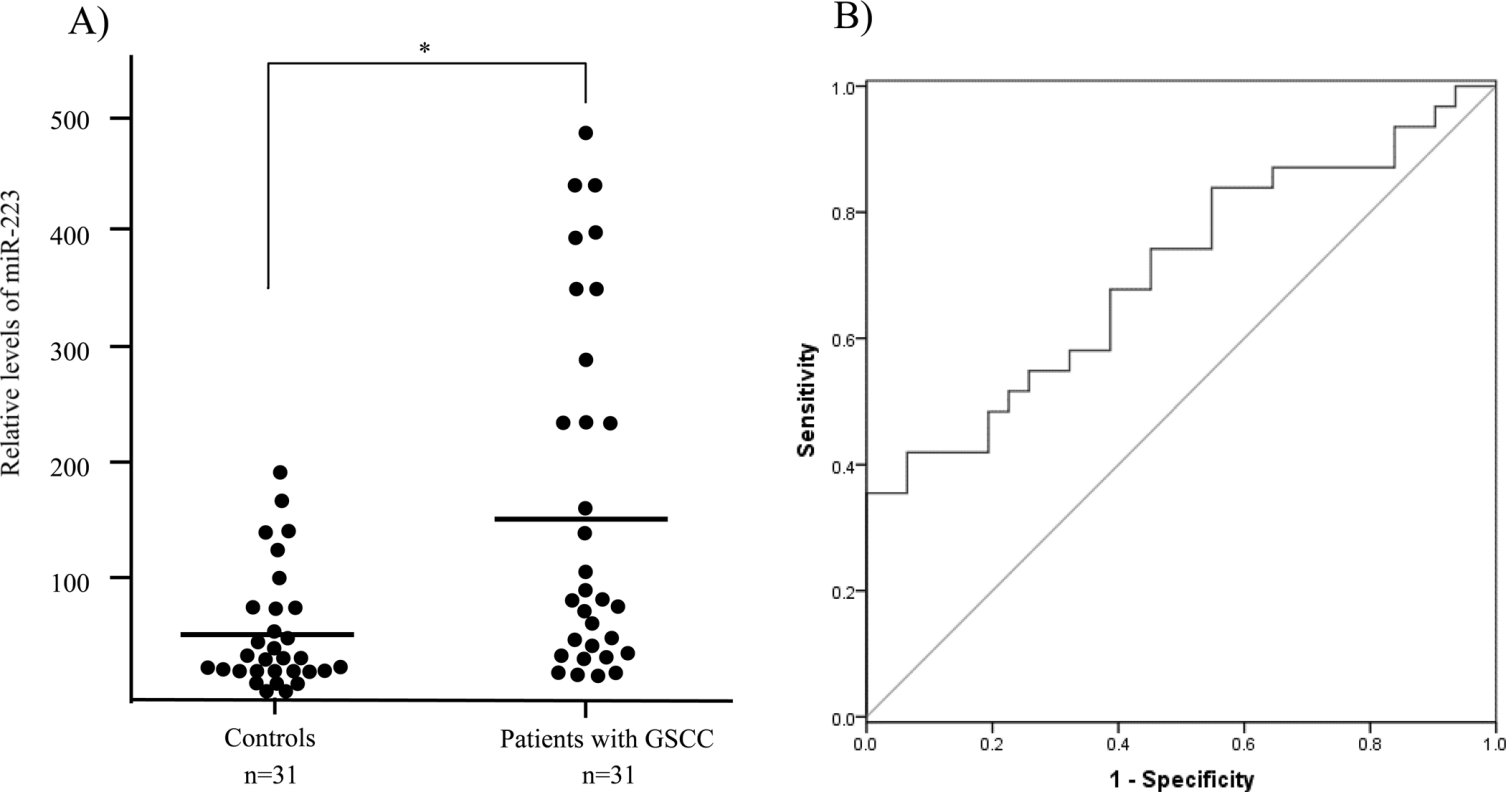
Validation and evaluation of plasma miR-223 as a biomarker of oral cancer. **(A)** The relative level of miR-223 expression in plasma from patients with GSCC and controls: *let7a* was used for normalization; *, *P* < 0.05. **(B)** Receiver operating characteristics (ROC) curve analysis using plasma miR-223 to differentiate oral cancer.

To validate findings related to circulating miR-223, a RT-qPCR assay was used to detect the level of miR-223 in plasma from 31 patients with GSCC and 31 age- and sex-matched healthy controls. Consistent with the results regarding the circulating miRNA profile, the level of miR-223 in plasma was significantly higher (> 2-fold, *P* < 0.05) in patients with GSCC than in controls (Fig 2A), suggesting that miR-223 may contribute to the development of oral cancer and that its circulating form may serve as a novel biomarker. However, the level of miR-223 in plasma was not found to be associated with tumor grade, tumor size, or the metastatic status of lymph nodes (data not shown). To evaluate the diagnostic value of plasma miR-223 in oral cancer, ROC curve analysis was performed (Fig 2B). The area under the curve (AUC) for plasma miR-223 was 0.703 (95% Cl: 0.573–0.834). At a cutoff value of 40.67, miR-223 had a sensitivity of 67.7% and a specificity of 61.3%.

### Up-regulation of miR-223 in adjacent noncancerous tissues rather than cancer tissues

**Fig 3:**
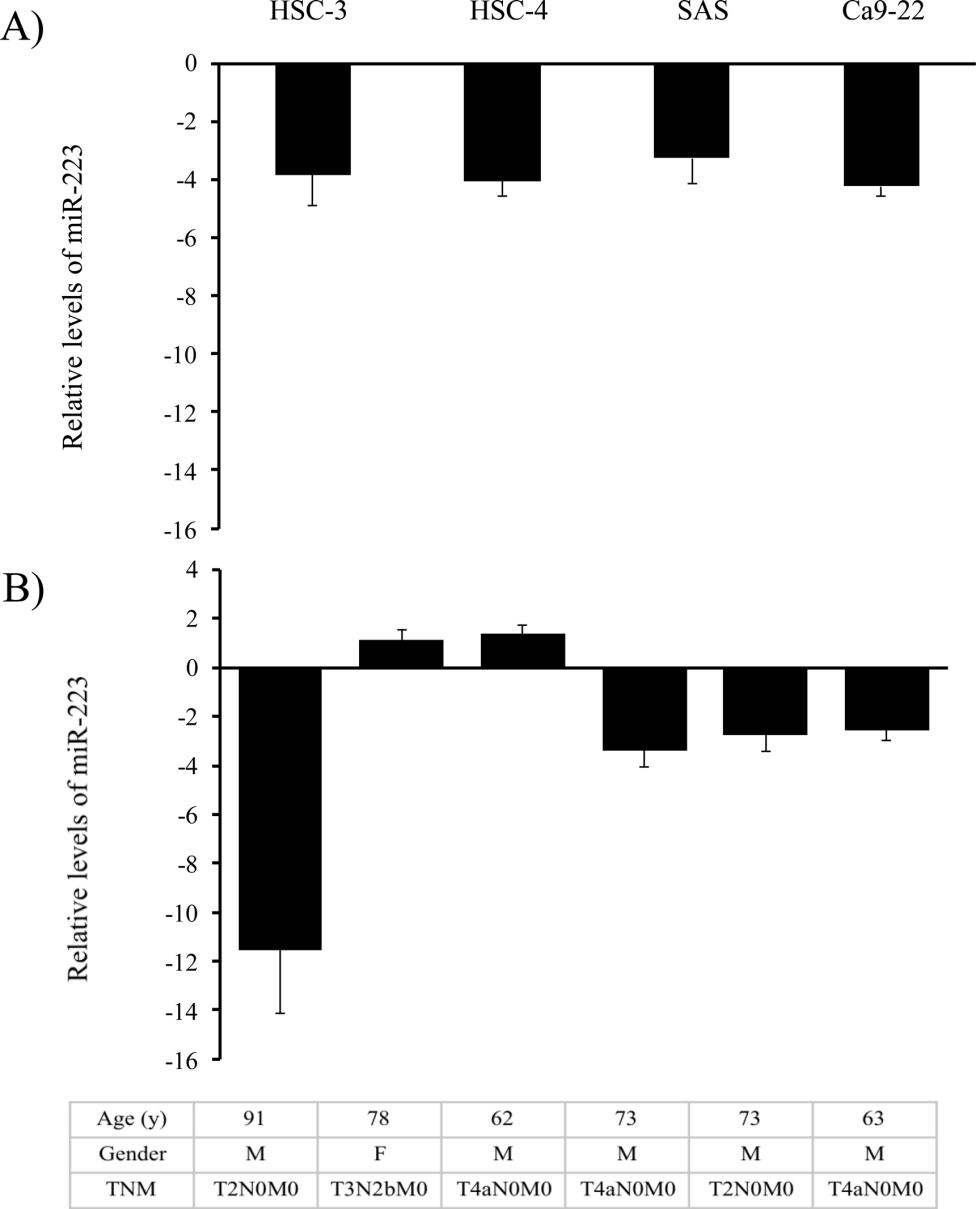
Relative levels of miR-223 expression in OSCC cell lines and tissues. **(A)** OSCC cell lines: Relative levels of miR-223 expression were evaluated using the equation–log_10_ 1/2^-⊿⊿Ct^ in which *ΔΔ*Ct = *Δ*Ct_cell line_^―^*Δ*Ct_normal.tissue._ The *Δ*Ct value of miR-223 in each sample was calculated by normalization with endogenous control RNU6B (*Δ*Ct = Ct_miRNA_- Ct_U6RNA_). **(B)** OSCC tissues: The upper panel shows the relative levels of miR-223 expression that were evaluated using the equation 2^―*ΔΔ*Ct^ when 2^―*ΔΔ*Ct^ ≥1 or -1/2^⊿⊿Ct^ when 2^―*ΔΔ*Ct^ < 1 in which *ΔΔ*Ct = *Δ*Ct_OSCC tissue_^―^*Δ*Ct_normal tissue_, and *Δ*Ct = Ct_miRNA_- Ct_U6RNA_. The lower panel shows the characteristics of patients from whom cancerous and adjacent non-cancerous tissues were tested. TNM: tumor node metastasis staging system.

To explore the potential sources of circulating miR-223 in patients with GSCC, the levels of miR-223 expression were first examined in a panel of human OSCC cell lines as well as in specimens of human normal oral mucosa. Surprisingly, miR-223 was expressed at vastly lower levels in all 4 oral cancer cell lines examined than in normal oral tissues (Fig 3A). When miR-223 was further examined in sets of GSCC and adjacent non-cancerous tissues from 6 patients with high levels of circulating miR-223, miR-223 was found to be down-regulated in 4 of the 6 tumor samples (66.67%; Fig 3B). In other words, up-regulation of miR-223 frequently occurred in adjacent normal tissues rather than in cancer tissues, implying that circulating miR-223 is probably released by noncancerous oral tissues and that miR-223 may have an important role in the development of oral cancer.

### The tumor-suppressing role of miR-223 on human GSCC cells

**Fig 4:**
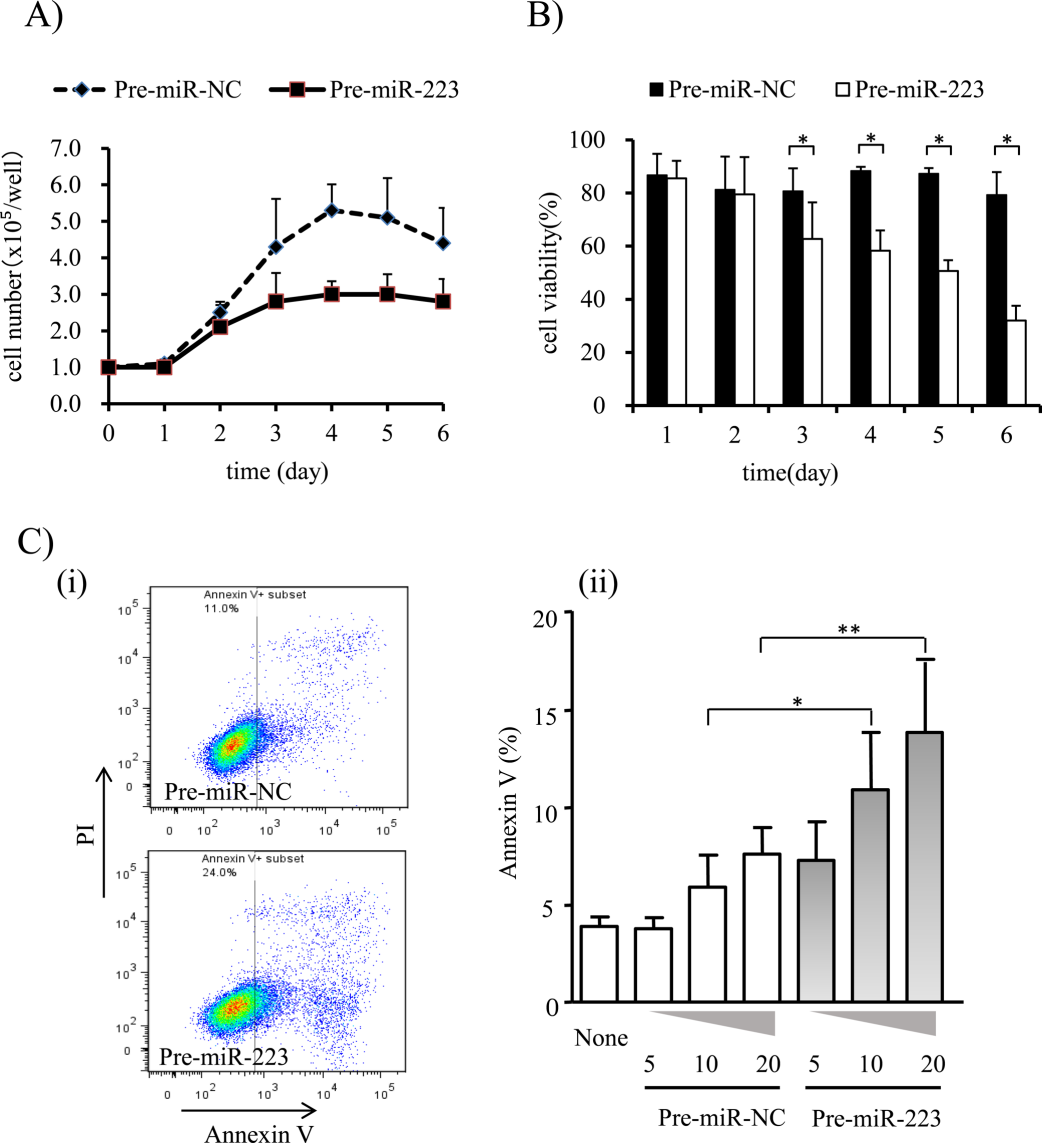
Effects of miR-223 on cell proliferation, viability, and apoptosis. **(A)** Growth curves of Ca9-22 cells transfected with Pre-miR-223 or Pre-miR-NC. **(B)** Viability of Ca9-22 cells transfected with Pre-miR-223 or Pre-miR-NC. Data are presented as the mean ± standard deviation (SD). **(C)** Augmentation of apoptosis in Ca9-22 cells transfected with Pre-miR-223. (i) Representative data from flow cytometry analysis. Ca9-22 cells were transfected with Pre-miR-223 or Pre-miR-NC. After incubation for 60 h, cells were analyzed with flow cytometry. (ii) Dose-response effect of miR-223 on apoptosis. Ca9-22 cells were treated with 5, 10, or 20 nM of Pre-miR-223 or Pre-miR-NC for 60 h, and the percentage of apoptotic cells (Annexin V %) was then measured. ‘None’ indicates the cells were not treated with any miRNAs. Data are presented as the mean ± standard error (n = 4). Each group was compared to ‘None’ (1-way ANOVA with post-hoc Dunnet’s test); *, *P* ≤ 0.05, and **, *P* ≤ 0.01.

To understand the effects of miR-223 on cell proliferation, apoptosis, and cell migration, a gain-of-function study was performed by transfecting miR-223 precursor into human GSCC Ca9-22 cells that exhibit low levels of endogenous miR-223 expression. As shown in Fig 4A and 4B, transfection with Pre-miR-223 significantly inhibited cell growth and decreased cell viability. A WST-1 assay 3 days after transfection further confirmed a reduction in cell proliferation and viability in cells transfected with Pre-miR-223 as compared to controls (3.0 ± 0.55 vs. 5.1 ± 1.1, *P* < 0.05) although marked morphological changes were not observed. To ascertain whether the reduced viability of cells transfected with Pre-miR-223 was due to apoptosis, annexin V staining was performed (Fig 4C). Sixty hours after transfection, miR-223-transfected cells showed a 2-fold increase in annexin V staining as compared to control cells (*P* < 0.05), indicating that miR-223 induced early apoptosis. A cell migration assay, however, revealed that the number of cells passing through the membrane did not differ significantly between Ca9-22 cells transfected with Pre-miR-223 and those transfected with Pre-miR-NC at 24 h or 48 h (S1 Fig). Taken together, these results suggest that miR-223 inhibits the development of oral cancer.

### Target genes of miR-223 in GSCC

**Fig 5:**
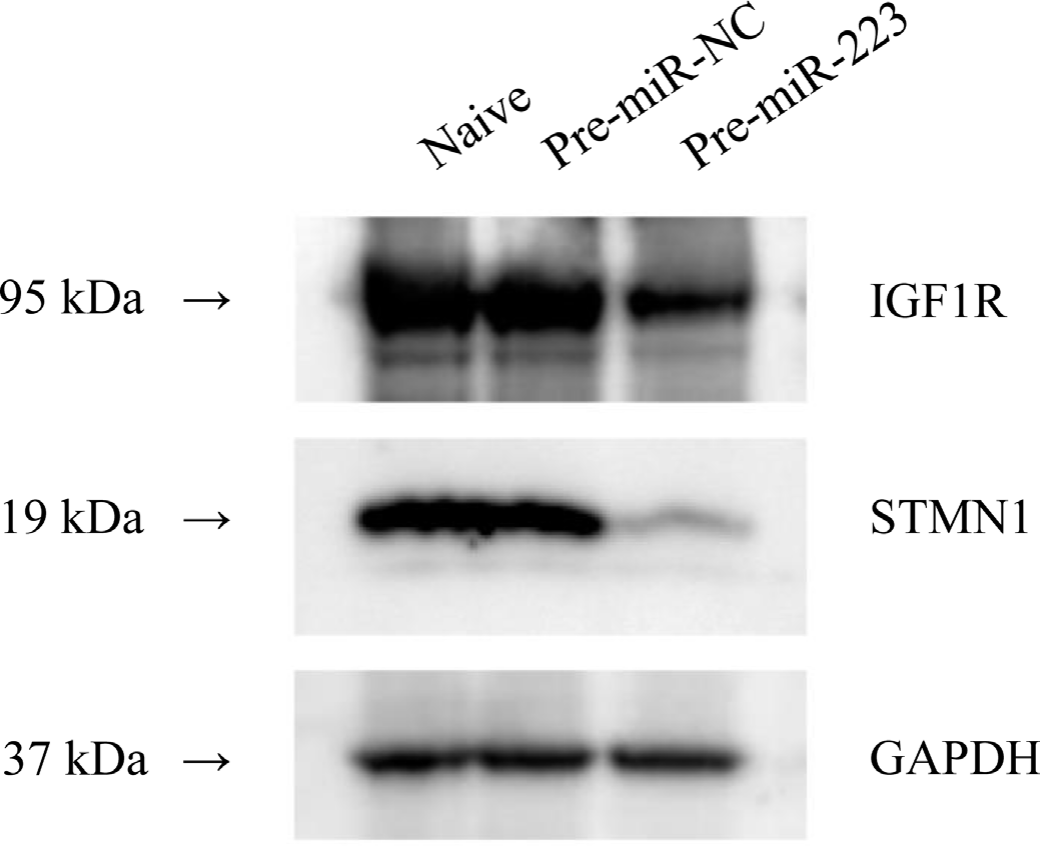
Immunoblotting of STMN1 and IGF1R in Ca9-22 cells transfected with Pre-miR-223 and Pre-miR-NC. Shown is a representative result from three independent experiments with Western blotting. GAPDH was used as a control.

The computational prediction algorithm miRWalk (http://zmf.umm.uni-heidelberg.de/apps/zmf/mirwalk2/) indicated that there were 90 potential target genes of miR-223 (S2 Table). When further functional annotation analysis was performed using the Database for Annotation, Visualization, and Integrated Discovery (DAVID) (https://david.ncifcrf.gov/home.jsp), nearly one-third of the predicted candidate genes were categorized as the apoptosis group. Two of these potential target genes, stathmin (STMN1) and the insulin-like growth factor 1 receptor (IGF1R), attracted attention because both have been reported to be the target genes of miR-223 in hepatocellular carcinoma [24] and cervical cancer [25]. Western blot analysis revealed a clear reduction in levels of STMN1 and IGF1R expression in Ca9-22 cells transfected with Pre-miR-223 in comparison to controls (Fig 5), suggesting that both are the targets of miR-223 in GSCC.

## Discussion

Since the discovery of the involvement of miRNAs in cancer development, remarkable progress has been made in understanding the biological role and clinical relevance of miRNAs in cancer [10]. Accumulated evidence has shown that circulating miRNAs can potentially be used to detect a wide range of different types of cancer and to provide therapeutic interventions [11, 26]. However, identifying a circulating miRNA with clinical value is still a time-consuming and challenging task [27, 28]. In the area of oral cancer, most if not all studies of circulating miRNA have focused primarily on intracellular miRNA dysregulation in surgically resected cancer tissues or cell lines, and those aberrantly expressed miRNAs were then used to screen for appropriate biomarkers in serum or plasma from patients [13, 15, 16, 29]. Because circulating miRNAs exist at very low concentrations in blood and do not always reflect their levels of expression in cancer tissue, only a few circulating miRNAs have been cited as potential biomarkers in oral cancer thus far [18, 30]. Recently, technological advances in high-throughput screening improved the sensitivity and accuracy with which circulating miRNAs are identified [31, 32]. The current study used a high-sensitivity and high-throughput array to determine the circulating miRNA profile for patients with GSCC. Results identified miR-223 as an up-regulated circulating miRNA with tumor-inhibiting action.

Profile analysis of 1,211 human miRNAs in plasma revealed that 20 miRNAs were significantly dysregulated (~2-fold) in patients with GSCC as compared to healthy individuals. Sixteen of these miRNAs were up-regulated and 4 were down-regulated. These numbers and patterns of circulating miRNA are similar to those reported by Ries et al., who investigated the global miRNA expression in whole blood samples from patients with OSCC in Europe [18]. Nevertheless, the 3 most prominently deregulated miRNAs that Ries et al. discovered, miR-186, miR-3651, and miR-494, were not among the 20 miRNAs identified in the current study. This disparity in results may be explained by a difference in the specimens analyzed since a recent study has found that blood cell miRNAs affect the analysis of circulating miRNAs [28]. Alternatively, another explanation could be because the current sample consisted only of GSCC while the study by Ries et al. included different types of oral cancer [18]. MacLellan et al. also examined the expression profiles of circulating miRNAs in the serum of patients with high-risk oral lesions (HRLs, oral cancer or carcinoma in situ) in North America [33]. Their study, however, showed an expression profile distinct from those revealed by Ries et al. [18] and us, One miRNA, miR-223, that was considerably downregulated in serum from their patients with HRLs [33], has been shown to be the most significantly upregulated miRNA in our profile analysis. These inconsistent results probably reflect the diverse sources (e.g., serum, plasma, whole blood sample) and sorts of analysis methods used in different studies (e.g., platforms, normalization methods), suggesting the need for further validation of the reported findings.

Because of its abundance and fold change, miR-223 was selected as a potential candidate in order to validate the current results of the high throughput array and identify a reliable and clinically useful biomarker for detection of oral cancer. As several studies have indicated that blood cells and hemoglobin can considerably influence levels of circulating miRNAs in plasma [28, 34], close attention has been paid to sample preparation, i.e. separating plasma within 4 h of blood collection and carefully avoiding hemolysis and platelet contamination. The current validation tests revealed that circulating miR-223 levels were significantly higher (~2-fold, *P* <0.05) in 31 patients with GSCC as compared to those in health individuals, confirming the results of the miRNA profile analysis. One might argue the data normalization used played a role. There is no standard endogenous control for real-time quantification of miRNA in plasma, let-7a was selected for data normalization because many studies of circulating miRNAs have suggested that let-7a is an optimal endogenous control [35, 36]. In their quantifying of circulating miRNA biomarkers in plasma via qRT-PCR, Tewari et al. added a non-human exogenous miRNA (cel-miR-39; a synthetic *C*. *elegans* miRNA) for data normalization [36]. Similarly, the current study added cel-miR-39 to paired plasma as a spiked-in control to verify whether let-7a was suitable for use as an endogenous control for normalization. The results derived from the two methods of normalization were highly similar (correlation coefficient = 0.74; data not shown). Therefore, there is no doubt that the level of circulating miR-223 is significantly upregulated in patients with GSCC.

When ROC curve analysis was used to evaluate the potential for circulating miR-223 to serve as a diagnostic marker of GSCC, miR-223 resulted in an AUC value of 0.73. This performance is very close to that of biomarkers that have recently been reported, e.g. plasma miR-196a [30] and salivary IL-8 [37]. In addition, miR-223 was able to distinguish patients with GSCC from health controls with a moderate level of sensitivity (67.7%) and specificity (61.3%). Those levels of sensitivity and specificity are similar to the levels of many other novel biomarkers for cancer diagnosis [6]. Moreover, those levels of sensitivity and specificity are far superior to the levels of sensitivity and specificity of oral cancer biomarkers, such as SCC antigen [38], that are currently in clinical use. These findings suggest that miR-223 may serve as a novel biomarker for diagnosing GSCC. An interesting question for the future is whether miR-223 can be incorporated into a panel with other circulating miRNA biomarkers such as miR-196a and miR-196b [30] to improve diagnostic accuracy. In addition, further studies are needed to determine if circulating miR-223 has clinical utility in terms of diagnosing oral cancer and determining its prognosis.

Although an increasing number of studies have investigated the potential clinical use of circulating miRNAs in oral cancer [26, 29], relatively little attention has been paid to their cellular origins [19]. Circulating miRNAs are generally thought to be released by cancer tissues [19, 26], either as cell death byproducts or as cell secretory components, although some studies have argued that circulating miRNAs might reflect a blood cell-based phenomenon rather than a cancer-specific origin [34]. Since the current plasma samples were prepared with great care to prevent cell component contamination, miR-223 was originally assumed to be released by cancer tissue. Nevertheless, quantitation of miR-223 expression in 4 oral cancer cell lines indicated that miR-223 was expressed at extremely low levels in GSCC cells. Of particular interest is the fact that the levels of miR-223 expression were significantly lower in cancer tissue than in surrounding normal tissues obtained from 4 of 6 patients with GSCC. In other words, miR-223 expression was up-regulated in normal tissues surrounding cancer. These findings suggest that normal rather than tumor tissue might contribute the high level of miR-223 in plasma from patients with oral cancer. Several pieces of evidence also support this contention. First, recent studies have indicated that some extracellular miRNAs may perform cell-cell communication [39]. For example, Kosaka et al. reported that some secretory miRNAs in normal prostate PNT-2 cells inhibit the proliferation of prostate cancer cells *in vitro* [40]. Second, a functional analysis of miR-223 using the oral cancer cell line Ca9-22 revealed that miR-223 acts as a tumor suppressor by inhibiting cell proliferation and inducing apoptosis, further implying that a high level of miR-223 in plasma from patients with GSCC is probably because normal tissue surrounding a tumor secretes miR-223 as a biological defense mechanism to inhibit tumor growth. Immunohistochemical analysis of miR-223 expression in tumors as well as in surrounding normal tissue would be required to confirm this hypothesis regarding the origins of circulating miR-223.

To the extent known, this study is the first to show that miR-223 functions as a tumor suppressor in GSCC, although a few previous studies have revealed that miR-223 can inhibit cell proliferation and migration in cervical cancer and hepatocellular carcinoma [24, 25]. In addition, the current study identified STMN1 and IGF1R as the target genes of miR-223 in GSCC cells. Based on these findings, miR-223 and its target genes may be potential molecular targets for development of new treatments for GSCC. Indeed, development of molecular targeted therapy based on specific miRNAs for several diseases including cancer is rapidly proceeding. For instance, phase III clinical trials of a treatment for HCV using a miR-122 inhibitor have begun [41]. In a preclinical study, treatment with miR-34 mimic inhibited prostate cancer stem cells and prevented metastases [42]. Moreover, a phase II study with IGF1R as a molecularly targeted drug to treat prostate cancer is being conducted [43]. Therefore, targeting miR-223 and/or its related genes may lead to a novel therapeutic option for patients with oral cancer who are unable to undergo surgery. To this end, the pressing task is to elucidate the role of miR-223 in the development of oral cancer *in vivo*.

## Conclusions

Microarray analysis and subsequent validation tests revealed that miR-223 is found in significantly high levels in plasma from patients with GSCC. Functional analysis showed that miR-223 acted as a tumor suppressor by inhibiting cell proliferation and inducing apoptosis. The current data suggest that circulating miR-223 is a novel diagnostic biomarker and therapeutic target for oral cancer.

## Supporting Information

S1 Fig Migration assay of Pre-miR-223- or Pre-miR-NC- transfected Ca9-22 cells. Representative images are shown (Diff-Quik staining, ×10).
(PDF)

S2 Fig Full-size scans of western blot membranes shown in Fig 5. (PDF)

S1 Table Clinical characteristics of 31 patients with GSCC. (XLSX)

S2 Table Candidate target genes of miR-223. (XLSX)
