# Supplementary material for: Circulating miR-223 in Oral Cancer: Its Potential as a Novel Diagnostic Biomarker and Therapeutic Target
Source: PLoS One. 2016 Jul 21;11(7):e0159693. doi: 10.1371/journal.pone.0159693 (PMC4956265; doi:10.1371/journal.pone.0159693)
Supplement: S1 Fig — Representative images are shown (Diff-Quik staining, ×10). (PDF) [file pone.0159693.s001.pdf]

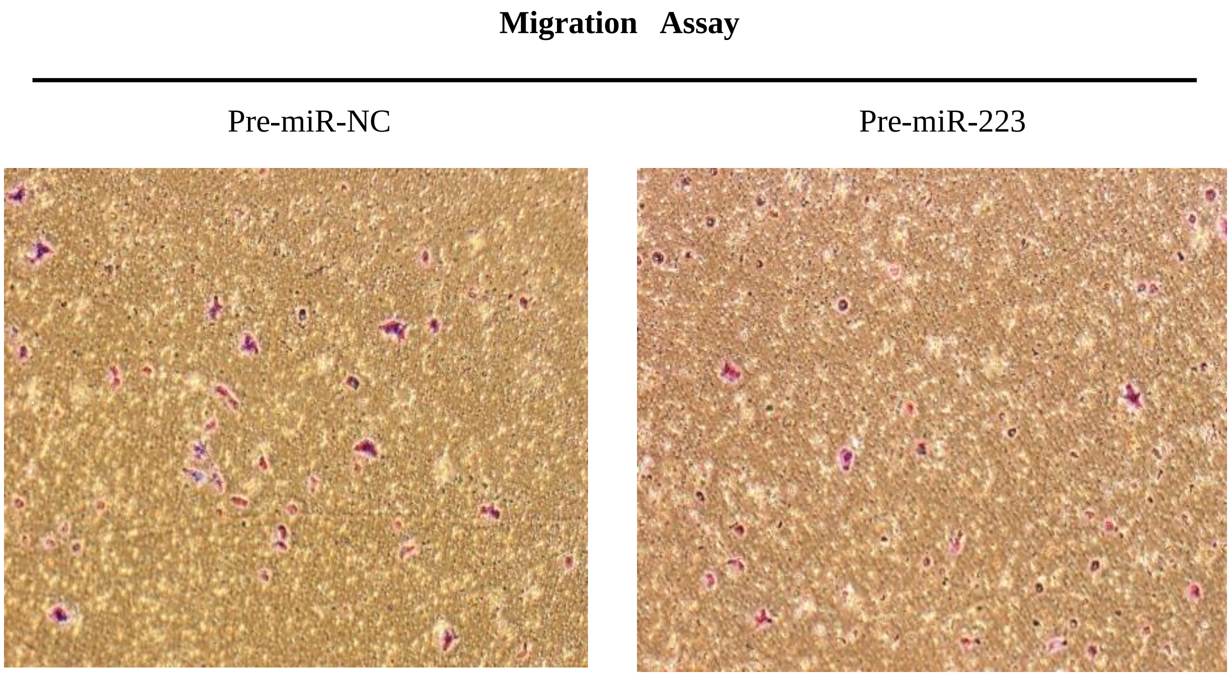

S1 Fig. Migration assay of Pre-miR-223- or Pre-miR-NC- transfected Ca9-22 cells.
