# Supplementary figures and images for: Circulating miR-223 in Oral Cancer: Its Potential as a Novel Diagnostic Biomarker and Therapeutic Target
Source: PLoS One. 2016 Jul 21;11(7):e0159693. doi: 10.1371/journal.pone.0159693 (PMC4956265; doi:10.1371/journal.pone.0159693)

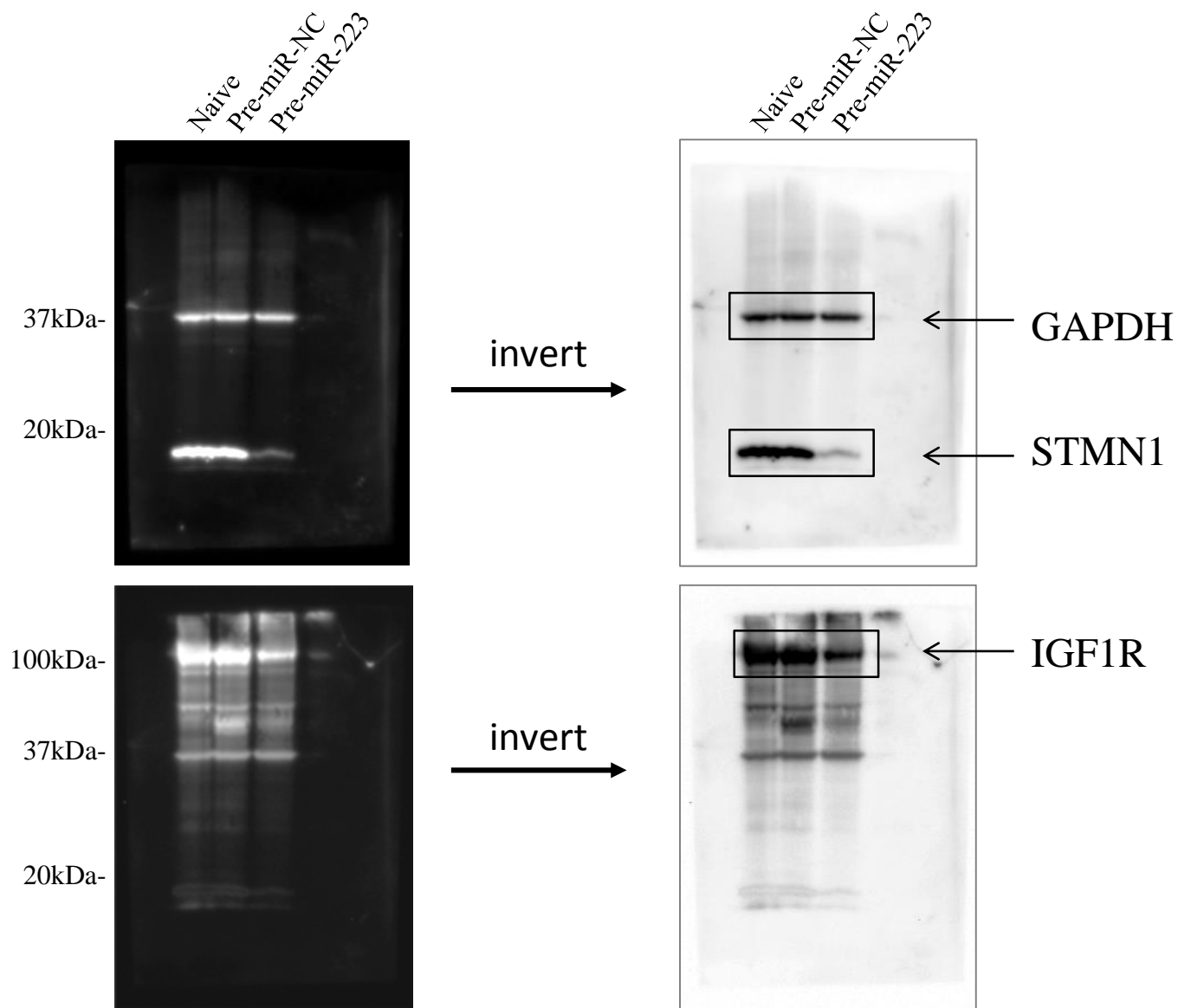

**S2 Fig. Full-size scans of western blot membranes shown in Fig 5.**

Supplement: S2 Fig — (PDF) [file pone.0159693.s002.pdf]
